# Childhood adversity and social anxiety among college students: the mediating roles of personal growth initiative and problematic social media use

**DOI:** 10.3389/fpsyg.2026.1761165

**Published:** 2026-05-07

**Authors:** Yan Li, Shunying Zhao, Yifu Qiu, Luyang Pi

**Affiliations:** 1 School of Marxism, Guangdong University of Petrochemical Technology, Maoming, China; 2 College of Education Science, Jiaying University, Meizhou, China; 3 Faculty of Liberal Arts and Law, Guangdong University of Petrochemical Technology, Maoming, China

**Keywords:** childhood adversity, college students, personal growth initiative, problematic social media use, social anxiety

## Abstract

Based on the life course theory, this study explores the relationship between childhood adversity and social anxiety among college students, as well as the roles of personal growth initiative and problematic social media use in this context. A sample survey was conducted on 909 college students, and the cross-sectional study results showed: (1) Childhood adversity and problematic social media use were significantly positively correlated with social anxiety among college students, while personal growth initiative was significantly negatively correlated with social anxiety. (2) Personal growth initiative and problematic social media use independently mediated the relationship between childhood adversity and social anxiety. (3) Personal growth initiative and problematic social media use played a parallel mediating role in the relationship between childhood adversity and social anxiety. This study revealed the mechanism of the association between childhood adversity and social anxiety among college students, expands the research perspective in related fields, and provided potential basis for a deeper understanding of the social anxiety problems among college students.

## Introduction

Social anxiety is one of the common interpersonal dilemmas faced by college students, which can bring a series of negative impacts on their daily life, such as lower academic achievement, limited social support system, and lack of work enthusiasm (Slivjak et al., 2024; Vilaplana-Pérez et al., 2021). Previous studies have found that childhood adversity may be one of the important factors affecting social anxiety among college students (Kumar and Marwaha, 2025). Childhood adversity, as a risk factor for the mental health of college students, can affect the development of their social skills, causing a lack of social interaction or avoidance, and leading to anxiety (McKee-Lopez et al., 2019; Wang et al., 2021). It may potentially hinder the career development abilities of college students, strain interpersonal relationships, and impair their mental health (Bellis et al., 2019).

However, the emergence and development of social anxiety among college students are not only related to objective factors such as various negative childhood experiences, but also to their internal psychological resources (e.g., personal growth initiative) and external behavioral factors (e.g., problematic social media use). Based on the theory of developmental trauma disorder, early trauma in individuals may weaken their ability to grow, triggering emotions such as anxiety and depression actively. To escape these emotions, individuals may compensate by using social media. Based on this, this study intends to explore the relationship between childhood adversity and social anxiety among college students, as well as the potential mechanisms by which personal growth initiative and problematic social media use contribute.

### Childhood adversity and social anxiety

Childhood adversity refers to stressful events or traumatic experiences, specifically such as violence, abuse, neglect, etc., suffered before the age of 18 that children encounter during their growth process, which may have a long-lasting negative impact on their physical and mental development (Goddard, 2021; McLaughlin, 2016). Adversity in childhood and early life can negatively affect the entire life cycle by affecting an individual’s physical and mental health (Bellis et al., 2019). Studies using self-report methods have found a very strong dose–response relationship between childhood adversity and mental health, as well as negative behaviors in adulthood. Especially when the cumulative number of adverse experiences reaches four or more, the impact of this dose–response relationship is significantly enhanced (Felitti et al., 1998; Hamby et al., 2021).

Social anxiety, as an essential indicator for measuring individual social adaptation, refers to the anxiety symptoms exhibited by individuals who fear or avoid social situations. It manifests as emotional discomfort, intense anxiety, and panic during face-to-face interactions with others, and sometimes even non-adaptive avoidance functioning. When this negative emotion reaches a severe level that affects normal social functioning, it is referred to as Social Anxiety Disorder (SAD) or Social Phobia (Morrison and Heimberg, 2013). According to the theory of developmental trauma disorders, as time progresses and individuals grow up, traumatic childhood experiences may be temporarily forgotten. However, the brain and body never truly erase their memories. Despite the passage of time, even minor dangerous stimuli can trigger overly intense adverse reactions, such as depression, anxiety, and risk-taking behaviors (Winterhalder and Leslie, 2002), disrupting an individual’s daily life. Statistics show that approximately one-third of global mental illnesses can be attributed to childhood adversity (McLaughlin et al., 2012). Findings from earlier research have revealed that childhood adversity can affect individuals’ self-evaluation, academic burnout, and sleep disorders, and lead to interpersonal difficulties (McKee-Lopez et al., 2019; Wang et al., 2021), potentially resulting in social anxiety.

### Mediating role of personal growth initiative

The impact of childhood adversity on mental health has a cumulative effect. Early trauma may lead to discomfort in adulthood through a “chain reaction”(e.g., educational interruption and economic difficulties). However, the impact can be altered by protective factors, such as social support and individual initiative (Elder, 1998). To reduce the impacts of childhood adversity on individual mental health, it implies that individuals need to actively grow, handle various adversities and trauma during their growth, and cope with multiple setbacks and challenges (Savickas, 1997). Personal growth Initiative is one of the positive internal psychological resources, which means the conscious and proactive efforts of individuals to enhance and improve their psychological tendencies during the process of development (Robitschek, 1998). Goodman et al. (2006) believe that an important reason for developing personal growth initiative is that most people will face many new problems and challenges during their growth process, and they need to prepare in advance to better cope with and adapt to these new changes. Studies have consistently shown that compared to people with low personal growth initiative, people with high personal growth initiative are psychologically healthier (Ogunyemi and Mabekoje, 2007; Robitschek and Keyes, 2009). A significant negative relationship exists between personal growth initiative and depression (Yang and Chang, 2016) as well as anxiety (Eslami, 2021). Hence, personal growth initiative may potentially contribute to the relationship between childhood adversity and social anxiety among college students.

### Mediating role of problematic social media use

With the rapid development of social technology, smartphones and social media are widely used. A series of problems caused by the unreasonable use of social media have attracted widespread social attention (Boer et al., 2021; Bozzola et al., 2022). Being overly dependent on social media may lead to problematic social media usage behavior. Unlike pathological internet addiction behavior, problematic social media use belongs to the category of general psychological issues, and its impact on individuals remains within a controllable range. It manifests as individuals engaging in prolonged and high-intensity social media usage, resulting in adverse physiological, psychological, and behavioral effects, which to some extent affect individuals’ lives and studies (Billieux et al., 2015). Previous studies have shown that childhood traumatic experiences not only have negative impacts on individuals’ physiology and psychology but also accompany internet addiction and mobile phone addiction behaviors (Dou et al., 2022; Liang et al., 2021; Xie et al., 2024). The theory of compensatory internet use (Kardefelt-Winther, 2014) suggests that people who experienced adversity in childhood may not have their psychological needs met, and social media, as a means of alleviating negative emotions during childhood adversity, is a compensation for unmet psychological needs. College students make up one of the leading groups of social media users, and the incidence of problematic social media usage behavior is relatively high (Spada, 2014), which is considered to carry the risk of developing harmful and addictive behaviors (Bargeron and Hormes, 2017; Makas et al., 2025). Data from a China Youth Daily survey in 2024 suggests that 86.6% of college students share their diverse lives through social media. Social media strengthens emotional and social connections among college students, playing a particular role in enhancing self-esteem and reducing negative emotions such as depression. However, being overly dependent on social media may lead to symptoms such as sleep disorders, poor academic performance, depression, anxiety, and loneliness (Beard and Wolf, 2001), reduce life satisfaction and happiness (Cerniglia et al., 2017), and affect the development of social skills among college students. It is speculated that problematic social media use may also play a role in the relationship between childhood adversity and social anxiety among college students.

**Figure 1:**
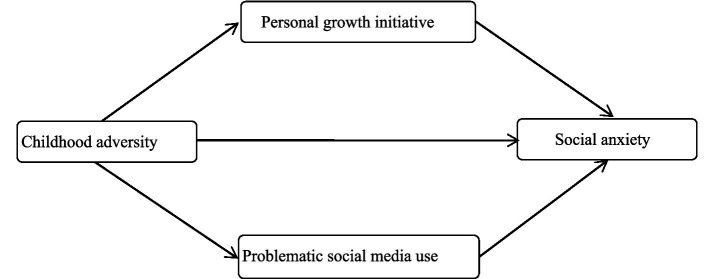
The hypothesized mediation model.

In conclusion, although numerous studies have explored the association between childhood adversity and social anxiety, there are still few discussions on the underlying mechanisms of this relationship. Particularly, insufficient attention has been paid to the combined effects of individual internal psychological resources (e.g., personal growth initiative) and external digital media behaviors (e.g., problematic social media use). Most of the existing studies only explore a single mediating path and lack the exploration of multi-path parallel mediating mechanisms. Based on this, this study intends to explore the relationship between childhood adversity and social anxiety among college students, as well as the potential mediating mechanisms of personal growth initiative and problematic social media usage in this process. The following hypotheses are proposed (see Figure 1).

*H1*: Childhood adversity is positively correlated with social anxiety in college students.

*H2*: Personal growth initiative and problematic social media use independently mediate the relationship between childhood adversity and social anxiety.

*H3*: Personal growth initiative and problematic social media use play a parallel mediating role between childhood adversity and social anxiety.

## Method

A convenience sampling method was adopted in data collection. In this study, the sample was from a questionnaire survey that was conducted on college students from two public universities in China through the online company Questionnaire Star between March and May 2025. Questionnaire Star is a widely used online platform with more than 19 years of experience in questionnaire surveys. To encourage participation, it has set up a reward mechanism and provided rewards for qualified individuals.

### Participants

A sample of 952 college students was recruited for this study. After data screening and quality control, 909 valid samples were ultimately included, with a valid questionnaire response rate of 95.48%. The final sample consists of 570 males (62.71%) and 339 females (37.29%); 594 freshmen (65.35%), 237 sophomores (26.07%), and 78 juniors (8.6%). All participants have signed informed consent forms and voluntarily participated in this survey.

### Measures

#### Childhood adversity

Childhood adversity was measured using the Scale of the Adverse Childhood Experiences Questionnaire revised by Finkelhor et al. (2015). It is mainly used to investigate family experiences under the age of 18, including 14 aspects such as physical abuse, emotional abuse, neglect, and family dysfunction. The answer is “yes” or “no,” with a score of 1 for “yes” and 0 for “no.” The maximum possible score is 14 points, and a higher score reflects more childhood adversity experienced by individuals. ACEQ-R is currently a comprehensive tool for studying childhood adverse experiences. For the current study, Cronbach’s alpha in this sample was 0.76.

#### Social anxiety

Social anxiety was measured by the Scale of Interaction Anxiousness Scale (Leary, 2010). The Scale is composed of 15 items, of which 11 items are positively scored, and 4 items are negatively scored. This scale involves subjective anxiety rather than specific external behaviors, a commonly used self-report measure to assess subjective social anxiety experience. There is strong evidence for the internal consistency and test–retest reliability of the Scale. With higher scores corresponding to higher levels of social anxiety. In this study, the Cronbach’s alpha of the scale was calculated as 0.85.

#### Personal growth initiative

The Personal Growth Initiative Inventory (PGI-II), developed by Robitschek et al. (2012), consists of 16 items and includes four dimensions, which are readiness for change, planning, resource utilization, and proactive behavior. The Scale scoring system ranging from “completely disagree (0)” to “completely agree (5),” and all items are scored positively. The higher the total score of each item, the higher the level of personal growth initiative. Different researchers verified the reliability and validity of the Scale in the research of American college students, Turkish college students, and Chinese students, indicating that the reliability and validity of the Scale are well-established. In this study, Cronbach’s alpha was calculated as 0.953.

#### Problematic social media use

Adopting the assessment questionnaire of problematic mobile social media usage (PMSMU-AQ) developed by Jiang Yongzhi in 2018, this questionnaire is generally used to assess the severity of symptoms linked to problematic social media usage, with a focus on examining individuals’ physiological, psychological, and behavioral aspects. PMSMU-AQ consists of 20 self-assessment items, including five factors which are increased stickiness, physiological damage, missed anxiety, cognitive failure, and guilt. The questionnaire adopts a 5-point scale, where 1 represents “completely inconsistent” and 5 represents “completely consistent.” The higher score on the scale means the more severe the tendency of adolescents’ problematic social media use behavior. Similarly, higher scores of each factor indicate a more severe behavioral tendency. In this study, Cronbach’s alpha of the questionnaire was 0.951.

### Data analysis

In this study, SPSS 27.0 was used to conduct descriptive statistics, correlation analysis, and common method bias tests on the data. The PROCESS macro plugin was employed to test the mediating effect.

## Results

### Common method bias

Since the data were collected through online self-report instruments, common method bias may exist. Therefore, to minimize the impact of common method bias, this study implemented necessary procedural controls, such as anonymous questionnaire completion and standardized administration. Additionally, Harman’s one-factor test (MacKenzie and Podsakoff, 2012) was used for statistical analysis. The findings suggested that there were 12 factors with eigenvalues greater than 1 and that the cumulative variance explained by the first factor was 20.08%, which did not reach the 40% threshold. Therefore, the results indicated that the common method bias was not serious in this research.

### Descriptive statistics and correlation analysis

As hypothesized, childhood adversity was negatively linked to personal growth initiative (*r* = −0.169, *p* < 0.001) and positively related to problematic social media use (*r* = 0.164, *p* < 0.001) and social anxiety (*r* = 0.166, *p* < 0.001). Personal growth initiative was negatively correlated with social anxiety (*r* = −0.149, *p* < 0.001), while problematic social media use was positively correlated with social anxiety (*r* = 0.420, *p* < 0.001) (see Table 1).

**Table 1:** Descriptive statistics and correlations for the variables.

Variables	*M*	SD	1	2	3	4
1 CA	1.033	1.753	1			
2 PGI	49.212	12.491	−0.169***	1		
3 PSMU	58.736	15.347	0.164***	−0.075*	1	
4 SA	46.350	8.701	0.166***	−0.149***	0.420***	1

CA, Childhood Adversity; PGI, Personal Growth Initiative; PSMU, Problematic Social Media Use; SA, Social Anxiety; *p < 0.05, **p < 0.01, ***p < 0.001; Two-tailed.

### Mediation analyses

The mediating effects of personal growth initiative and problematic social media use between childhood adversity and social anxiety were tested using Model 4 from the PROCESS plugin of the SPSS macro program developed by Hayes. First, childhood adversity was taken as the independent variable, and social anxiety as the outcome variable. Then, personal growth initiative and problematic social media use were sequentially tested as mediating variables. The results indicated that childhood adversity was significantly negatively associated with personal growth initiative (*β* = −1.205, *p* < 0.001), significantly positively associated with problematic social media use (*β* = 1.435, *p* < 0.001), and social anxiety (*β* = 0.413, *p* < 0.01). Personal growth initiative was significantly negatively associated with social anxiety (*β* = −0.073, *p* < 0.001), and problematic social media use was significantly positively associated with social anxiety (*β* = 0.226, *p* < 0.001). The results were displayed in Table 2.

**Table 2:** Testing for the mediation model.

Outcome variables	Independent variables	*R*	*R* ^2^	*F*	*β*	*t*
PGI (Model 1)	CA	0.169	0.029	26.668	−1.205	−5.164***
PSMU (Model 2)	CA	0.164	0.027	25.038	1.435	5.004***
SA (Model 3)	CA	0.443	0.197	73.861	0.413	2.721**
	PGI				−0.073	−3.474***
	PSMU				0.226	13.171***

*p < 0.05, **p < 0.01; ***p < 0.001.

**Figure 2:**
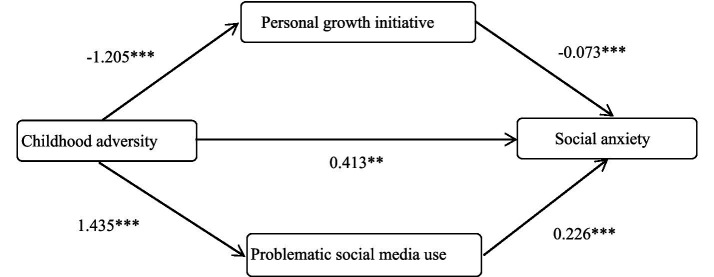
Results of the mediation model.

The results based on cross-sectional data indicate that personal growth initiative and problematic social media use play a statistically significant mediating role in the relationship between childhood adversity and social anxiety among college students. Among them, the direct path effect of childhood adversity and social anxiety was 0.413, accounting for 50% of the total effect, and the 95%CI did not include 0, indicating that the effect of this direct path reached a significant level. The mediation effects consist of indirect effects generated by the following two paths: Path 1, consisting of CA → PGI → SA, with an effect value of 0.088, accounting for 8.8% of the total effect, and 95%CI does not include 0, indicating that the indirect effect generated by this path is significant; Path 2, consisting of CA → PSMU→SA, with an effect value of 0.324, accounting for 32.4% of the total effect, and the 95%CI does not include 0, indicating that the indirect effect generated by this path reaches a significant level too. In addition, the study found that the mediation effect of PGI was significantly lower than that of PSMU (Effect = –0.236, 95% CI [–0.399, –0.090]). The results were displayed in Table 3 and Figure 2.

**Table 3:** Mediating effect analysis.

Model pathways	Effect	SE	Bootstrap95%CI	
			Lower	Upper
Direct effect				
CA → SA	0.413	0.152	0.115	0.711
Indirect effect				
CA → PGI → SA	0.088	0.035	0.026	0.165
CA → PSMU→SA	0.324	0.070	0.192	0.467
Total effects	0.826	0.163	0.507	1.145

SE, standard error; CI, confidence interval.

## Discussion

Although there have been many studies exploring the relationship between childhood adversity and mental health, there is still limited research from the perspective of life course theory that investigates the mechanism linking childhood adversity and social anxiety among college students. Therefore, this cross-sectional study explores how childhood adversity is associated with social anxiety among college students, as well as the roles played by personal growth initiative and problematic social media use. The results indicated that childhood adversity and problematic social media use were significantly positively correlated with social anxiety, whereas personal growth initiative was negatively correlated with social anxiety. Further mediation model analysis revealed that personal growth initiative and problematic social media use mediated the relationship between childhood adversity and social anxiety.

The study found a significant positive correlation between childhood adversity and social anxiety in college students, which was consistent with previous research (Carr et al., 2018; Poole et al., 2018), supporting H1. The theory of developmental trauma disorder suggests that childhood traumatic experiences are important risk factors for individual mental health and can have profound and lasting effects on the social, emotional, cognitive, and behavioral patterns of college students. Childhood adversity, such as physical and emotional abuse or neglect, peer bullying or harm, encouragement or exclusion, low socio-economic status, etc., may lead to more negative self-evaluation among college students, resulting in social anxiety, social withdrawal, and avoidance behaviors.

The study showed that personal growth initiative and problematic social media use independently mediated the relationship between childhood adversity and social anxiety, which supports H2. Previous studies have indicated that personal growth initiative can predict mental health (Ayub and Iqbal, 2012; Weigold et al., 2020). People with high personal growth initiative will be more proactive in seeking psychological help (Danitz et al., 2018; Seidman et al., 2022) and reduce pain and discomfort after psychotherapy by enhancing personal growth initiative (Weigold et al., 2018). Danese and McEwen (2012) explained this result from a neurophysiological perspective, suggesting that childhood adversity experiences may lead to structural and functional abnormalities in stress-related brain regions, affecting individual brain development and maturity, impairing emotional regulation abilities, and ultimately increasing the risk of depression and anxiety in adolescents. The self-determination theory (Deci and Ryan, 2012) posits that individuals possess an innate potential for growth and development, enabling them to flexibly control their interactions with the environment and freely choose their actions. When this control orientation originates from within, the pursuit of self-determination constitutes the internal motivation of human behavior. Personal growth initiatives encourage individuals to seek experiences that foster self-growth and personal improvement actively. Childhood adversity experiences are events that have already occurred and cannot be changed. Still, individuals can actively grow by changing their cognition and behavior, thereby reducing the psychological impact of childhood adversity on their future development.

Overuse of online social media can damage students’ academic performance and social interaction, leading to isolation (Bekir and Eldeleklioglu, 2023), anxiety and depression symptoms (Sitorus et al., 2020), and affecting students’ overall mental health (Makas et al., 2025). Childhood adversity may disrupt an individual’s cognitive function, interpersonal relationship patterns, and self-regulation abilities, leading to a series of maladaptive behaviors in adulthood, including problematic social media use. Individuals who have experienced childhood adversity and engage in negative interactions and social comparisons through social media may lead to higher levels of depression and anxiety symptoms (Seabrook et al., 2016).

The study also found that personal growth initiative and problematic social media use play a parallel mediating role between childhood adversity and social anxiety in college students, which supports H3. The life course theory emphasizes that the psychological development of an individual is the result of the interaction between the individual and the environment (Elder, 1998; Zhang et al., 2024). Personal growth initiative as an internal driving force for development is influenced by early childhood experiences, and the level of personal growth initiative will affect the individual’s subsequent psychological adaptation in turn. And problematic social media use will affect an individual’s real interpersonal communication, leading to social avoidance and anxiety. Therefore, personal growth initiative may play a protective role between childhood adversity and college students’ social anxiety, while problematic social media use may act as a risky factor. The parallel mediating path of personal growth initiative and problematic social media use in this study further validates that the association between childhood adversity and college students’ social anxiety is not the result of a single factor, but is formed through the joint effect of multiple factors, such as individual internal psychological resources and external behavioral choices. It must be clearly stated that the results of this study are based on the potential mechanisms underlying statistical correlations, rather than causal inferences. Further verification through tracking or experimental design will be necessary in the future.

It should be noted that in the parallel path model, the mediating effect of problematic social media use is significantly higher than that of personal growth initiative. This suggests that problematic social media use may play a more dominant role in the association between childhood adversity and social anxiety. The compensatory network use theory posits that the internet can provide individuals with immediate emotional satisfaction (Chen et al., 2026), but it cannot truly solve the individual’s real problems. The pressure and negative emotions in reality will make individuals more prone to problematic social media use, exacerbating negative social comparisons and negative self-evaluations on social media (Gruebner et al., 2021), and thereby leading to higher levels of social anxiety. Childhood adversity is one of the sources of individual stress and negative emotions (Qiao et al., 2023). Individuals who experience childhood adversity may be more prone to problematic social media use and develop higher levels of social anxiety. Personal growth initiative has an internally slow impact on individual psychological development. Therefore, the mediating effect of problematic social media use between childhood adversity and social anxiety is significantly greater than that of personal growth initiative. Additionally, there is a weak correlation between personal growth initiative and problematic social media use, thus failing to play a chain mediating role between childhood adversity and social anxiety among college students. The study speculates that college students with high personal growth initiative may use social media more as a way for learning or acquiring skills, offsetting the negative effects of problematic social media use, such as escaping reality or killing time. At the same time, college students with high personal growth initiative may have stronger self-control and goal management abilities, and even if they occasionally overuse social media, they can quickly reduce negative impacts through self-regulation.

### Limitations and future directions

Although this study has certain theoretical and practical significance, there are still some limitations that need attention. Firstly, social anxiety among college students from different cultural backgrounds may have cultural specificity. The sample of this study is limited to college students from two undergraduate universities in China, and there is a lack of cross-cultural samples of college students. This may affect the generalizability of the research conclusions. In the future, multi-cultural samples can be adopted to verify the conclusions of this study. Secondly, reliance on self-report methods may lead to measurement errors. In the future, other methods (e.g., peer assessment) can be used to overcome this limitation. Finally, this study only used cross-sectional data for analysis and cannot make causal inferences. In the future, longitudinal tracking or experimental research methods can be considered to reveal the mechanism of social anxiety among college students.

## Conclusion

This study proposed a parallel mediation model, breaking through the limitation of previous research that only focused on a single mediation path. It demonstrated that personal growth initiative and problematic social media use could, respectively, serve as parallel protective and risky paths, jointly explaining the association between childhood adversity and college students’ social anxiety. These findings further enrich the research on association models of social anxiety and potential basis for a deeper understanding of the social anxiety issues among college students.

## Data Availability

The raw data supporting the conclusions of this article will be made available by the authors, without undue reservation.

## References

[ref1] Ayub N. Iqbal S. (2012). The relationship of personal growth initiative, psychological well-being, and psychological distress among adolescents. J. Teach. Educ. 1, 101–107.

[ref2] Bargeron A. H. Hormes J. M. (2017). Psychosocial correlates of internet gaming disorder: psychopathology, life satisfaction, and impulsivity. Comput. Human Behav. 68, 388–394. doi: 10.1016/j.chb.2016.11.029

[ref3] Beard K. W. Wolf E. M. (2001). Modification in the proposed diagnostic criteria for internet addiction. Cyberpsychol. Behav. 4, 377–383. doi: 10.1089/109493101300210286, 11710263

[ref4] Bekir S. Eldeleklioglu J. (2023). School belongingness, peer relations, and insomnia as predictors of middle school pupils' problematic online gaming. J. Psychiatry Clin. Psychol. 23, 91–97. doi: 10.15557/PIPK.2023.0012

[ref5] Bellis M. A. Hughes K. Ford K. Rodriguez G. R. Sethi D. Passmore J. (2019). Life course health consequences and associated annual costs of adverse childhood experiences across Europe and North America: a systematic review and meta-analysis. Lancet Public Health 4, e517–e528. doi: 10.1016/S2468-2667(19)30145-8, 31492648 PMC7098477

[ref6] Billieux J. Maurage P. Lopez-Fernandez O. Kuss D. J. Griffiths M. D. (2015). Can disordered mobile phone use be considered a behavioral addiction? An update on current evidence and a comprehensive model for future research. Curr. Addict. Rep. 2, 156–162. doi: 10.1007/s40429-015-0054-y

[ref7] Boer M. Stevens G. W. Finkenauer C. de Looze M. E. van den Eijnden R. J. (2021). Social media use intensity, social media use problems, and mental health among adolescents: investigating directionality and mediating processes. Comput. Human Behav. 116:106645. doi: 10.1016/j.chb.2020.106645

[ref8] Bozzola E. Spina G. Agostiniani R. Barni S. Russo R. Scarpato E. . (2022). The use of social media in children and adolescents: scoping review on the potential risks. Int. J. Environ. Res. Public Health 19:9960. doi: 10.3390/ijerph19169960, 36011593 PMC9407706

[ref9] Carr A. Duff H. Craddock F. (2018). A systematic review of the outcome of child abuse in long-term care. Trauma Violence Abuse 21, 660–677. doi: 10.1177/1524838018789154, 30033824

[ref10] Cerniglia L. Zoratto F. Cimino S. Laviola G. Ammaniti M. Adriani W. (2017). Internet addiction in adolescence: neurobiological, psychosocial and clinical issues. Neurosci. Biobehav. Rev. 76, 174–184. doi: 10.1016/j.neubiorev.2016.12.024, 28027952

[ref11] Chen S. Yang L. Qu Y. Zhou N. (2026). Does emotional distress tolerance negatively predict problematic smartphone use or vice versa? Evidence from a longitudinal study and a daily diary study. Addict. Behav. 172:108531. doi: 10.1016/j.addbeh.2025.108531, 41167017

[ref12] Danese A. McEwen B. S. (2012). Adverse childhood experiences, allostasis, allostatic load, and age-related disease. Physiol. Behav. 106, 29–39. doi: 10.1016/j.physbeh.2011.08.019, 21888923

[ref13] Danitz S. B. Orsillo S. M. Beard C. Björgvinsson T. (2018). The relationship between personal growth and psychological functioning in individuals treated in a partial hospital setting. J. Clin. Psychol. 74, 1759–1774. doi: 10.1002/jclp.22627, 29696645

[ref14] Deci E. L. Ryan R. M. (2012). Self-determination theory Handbook of Theories of Social Psychology, Vol 1, eds. Van Lange P. A. M. Kruglanski A. W. Tory Higgins E. (London: SAGE publications Ltd).

[ref15] Dou K. Feng X.-K. Wang L.-X. Li J.-B. (2022). Longitudinal association between parental involvement and internet gaming disorder among Chinese adolescents: consideration of future consequences as a mediator and peer victimization as a moderator. J. Behav. Addict. 11, 820–830. doi: 10.1556/2006.2022.00056, 35994364 PMC9872523

[ref16] Elder G. H. (1998). The life course as developmental theory. Child Dev. 69, 1–12. doi: 10.1111/j.1467-8624.1998.tb06128.x, 9499552

[ref17] Eslami H. (2021). Personal Growth Initiative as a Parsimonious and Modifiable Predictor of Treatment Outcome in a Clinical Sample of University Students, (Doctoral Dissertation). Lubbock, TX: Texas Tech University.

[ref18] Felitti V. J. Anda R. F. Nordenberg D. Williamson D. F. Spitz A. M. Edwards V. . (1998). Relationship of childhood abuse and household dysfunction to many of the leading causes of death in adults: the adverse childhood experiences (ACE) study. Am. J. Prev. Med. 14, 245–258. doi: 10.1016/s0749-3797(98)00017-8, 9635069

[ref19] Finkelhor D. Shattuck A. Turner H. Hamby S. (2015). A revised inventory of adverse childhood experiences. Child Abuse Negl. 48, 13–21. doi: 10.1016/j.chiabu.2015.07.011, 26259971

[ref20] Goddard A. (2021). Adverse childhood experiences and trauma-informed care. J. Pediatr. Health Care 35, 145–155. doi: 10.1016/j.pedhc.2020.09.001, 33129624

[ref21] Goodman J. Schlossberg N. K. Anderson M. L. (2006). Counseling Adults in Transition: Linking Practice with Theory. New York: Springer Publishing Company.

[ref22] Gruebner O. Kim H. Schlicht R. Schardt M. Florack A. (2021). The contributions of social comparison to social network site addiction. PLoS One 16:e0257795. doi: 10.1371/journal.pone.0257795 34710108 PMC8553147

[ref23] Hamby S. Elm J. H. Howell K. H. Merrick M. T. (2021). Recognizing the cumulative burden of childhood adversities transforms science and practice for trauma and resilience. Am. Psychol. 76, 230–242. doi: 10.1037/amp0000763, 33734791

[ref24] Kardefelt-Winther D. (2014). A conceptual and methodological critique of internet addiction research: towards a model of compensatory internet use. Comput. Hum. Behav. 31, 351–354. doi: 10.1016/J.CHB.2013.10.059

[ref25] Kumar G. Marwaha E. B. (2025). Tracing the impact of childhood adversity on social anxiety in late adolescence: the moderating role of social support and coping strategies. Int. J. Adolesc. Med. Health 37, 279–287. doi: 10.1515/ijamh-2025-0040, 40996437

[ref26] Leary M. R. (2010). Social anxiousness: the construct and its measurement. J. Pers. Assess. 47, 66–75. doi: 10.1207/s15327752jpa4701_8, 6834234

[ref27] Liang H.-Y. Zhang B. Jiang H.-B. Zhou H.-L. (2021). Adult attachment: its mediation role on childhood trauma and mobile phone addiction. J. Psychol. Afr. 31, 369–374. doi: 10.1080/14330237.2021.1952706

[ref28] MacKenzie S. B. Podsakoff P. M. (2012). Common method bias in marketing: causes, mechanisms, and procedural remedies. J. Retail. 88, 542–555. doi: 10.1016/j.jretai.2012.08.001

[ref29] Makas S. Batmaz H. Aslan E. Çelik E. (2025). The role of mental health continuum and online gaming addiction as mediators in the relationship between school belongingness and meaningful school experience among adolescents. Curr. Psychol. 44, 12466–12478. doi: 10.1007/s12144-025-08069-3

[ref30] McKee-Lopez G. Robbins L. Provencio-Vasquez E. Olvera H. (2019). The relationship of childhood adversity on burnout and depression among BSN students. J. Prof. Nurs. 35, 112–119. doi: 10.1016/j.profnurs.2018.09.008, 30902402

[ref31] McLaughlin K. A. (2016). Future directions in childhood adversity and youth psychopathology. J. Clin. Child Adolesc. Psychol. 45, 361–382. doi: 10.1080/15374416.2015.1110823, 26849071 PMC4837019

[ref32] McLaughlin K. A. Green J. G. Gruber M. J. Sampson N. A. Zaslavsky A. M. Kessler R. C. (2012). Childhood adversities and first onset of psychiatric disorders in a national sample of US adolescents. Arch. Gen. Psychiatry 69, 1151–1160. doi: 10.1001/archgenpsychiatry.2011.2277, 23117636 PMC3490224

[ref33] Morrison A. S. Heimberg R. G. (2013). Social anxiety and social anxiety disorder. Annu. Rev. Clin. Psychol. 9, 249–274. doi: 10.1146/annurev-clinpsy-050212-185631, 23537485

[ref34] Ogunyemi A. O. Mabekoje S. O. (2007). Self-efficacy, risk-taking behavior and mental health as predictors of personal growth initiative among university undergraduates. Electron. J. Res. Educ. Psychol. 5, 349–362.

[ref35] Poole J. C. Dobson K. S. Pusch D. (2018). Do adverse childhood experiences predict adult interpersonal difficulties? The role of emotion dysregulation. Child Abuse Negl. 80, 123–133. doi: 10.1016/j.chiabu.2018.03.006, 29604503

[ref36] Qiao Z. Lafit G. Lecei A. Achterhof R. Kirtley O. J. Hiekkaranta A. P. . (2023). Childhood adversity and emerging psychotic experiences: a network perspective. Schizophr. Bull. 50, 47–58. doi: 10.1093/schbul/sbad079, 37318106 PMC10754171

[ref37] Robitschek C. (1998). Personal growth initiative: the construct and its measure. Meas. Eval. Couns. Dev. 30, 183–198. doi: 10.1080/07481756.1998.12068941

[ref38] Robitschek C. Ashton M. W. Spering C. C. Geiger N. Byers D. Schotts G. C. . (2012). Development and psychometric evaluation of the personal growth initiative scale–II. J. Couns. Psychol. 59, 274–287. doi: 10.1037/a0027310, 22352950

[ref39] Robitschek C. Keyes C. L. (2009). Keyes's model of mental health with personal growth initiative as a parsimonious predictor. J. Couns. Psychol. 56, 321–329. doi: 10.1037/a0013954

[ref40] Savickas M. L. (1997). Career adaptability: an integrative construct for life-span, life-space theory. Career Dev. Q. 45, 247–259. doi: 10.1002/j.2161-0045.1997.tb00469.x

[ref41] Seabrook E. M. Kern M. L. Rickard N. S. (2016). Social networking sites, depression, and anxiety: a systematic review. JMIR Ment. Health 3:e5842. doi: 10.2196/mental.5842, 27881357 PMC5143470

[ref42] Seidman A. J. Crick K. A. Wade N. G. (2022). Personal growth initiative, mental health stigma, and intentions to seek professional psychological help: a model extension. Stigma Health 7, 142–151. doi: 10.1037/sah0000369

[ref43] Sitorus N. Arfines P. P. Suryaputri I. Y. (2020). Relationship between online game addiction with depression in adolescents from 6 high schools in Indonesia. Global J. Health Sci. 12, 43–52. doi: 10.5539/gjhs.v12n12p43

[ref44] Slivjak E. T. Al Majid F. Wrigley J. Russell S. Zielony L. Arch J. J. (2024). Self-compassion and social anxiety: a scoping review. Mindfulness 15, 2448–2472. doi: 10.1007/s12671-024-02450-2

[ref45] Spada M. M. (2014). An overview of problematic internet use. Addict. Behav. 39, 3–6. doi: 10.1016/j.addbeh.2013.09.007, 24126206

[ref46] Vilaplana-Pérez A. Pérez-Vigil A. Sidorchuk A. Brander G. Isomura K. Hesselmark E. . (2021). Much more than just shyness: the impact of social anxiety disorder on educational performance across the lifespan. Psychol. Med. 51, 861–869. doi: 10.1017/S0033291719003908, 31907098 PMC8108394

[ref47] Wang Y. Hu X. Han J. Scalabrini A. Hu Y. Hu Z. . (2021). Time is of essence-abnormal time perspectives mediate the impact of childhood trauma on depression severity. J. Psychiatr. Res. 137, 534–541. doi: 10.1016/j.jpsychires.2020.10.039, 33153758

[ref48] Weigold I. K. Boyle R. A. Weigold A. Antonucci S. Z. Mitchell H. B. Martin-Wagar C. A. (2018). Personal growth initiative in the therapeutic process: an exploratory study. Couns. Psychol. 46, 481–504. doi: 10.1177/0011000018774541

[ref49] Weigold I. K. Weigold A. Russell E. J. Wolfe G. L. Prowell J. L. Martin-Wagar C. A. (2020). Personal growth initiative and mental health: a meta-analysis. J. Couns. Dev. 98, 376–390. doi: 10.1002/jcad.12340

[ref50] Winterhalder B. Leslie P. (2002). Risk-sensitive fertility: the variance compensation hypothesis. Evol. Hum. Behav. 23, 59–82. doi: 10.1016/S1090-5138(01)00089-7

[ref51] Xie Y. Shen Y. Wu J. (2024). Cumulative childhood trauma and mobile phone addiction among Chinese college students: role of self-esteem and self-concept clarity as serial mediators. Curr. Psychol. 43, 5355–5363. doi: 10.1007/s12144-023-04734-7, 37359599 PMC10172069

[ref52] Yang H. Chang E. C. (2016). Is the PGIS-II redundant with the hope scale?: evidence for the utility of the PGIS-II in predicting psychological adjustment in adults. Pers. Individ. Differ. 94, 124–129. doi: 10.1016/j.paid.2016.01.019

[ref53] Zhang Y. Shaojun C. Akintunde T. Y. Okagbue E. F. Isangha S. O. Musa T. H. (2024). Life course and mental health: a thematic and systematic review. Front. Psychol. 15:1329079. doi: 10.3389/fpsyg.2024.1329079, 39309150 PMC11412817

